# Increased circulating microRNA-122 is associated with mortality and acute liver injury in the acute respiratory distress syndrome

**DOI:** 10.1186/s12871-018-0541-5

**Published:** 2018-06-23

**Authors:** Tim Rahmel, Katharina Rump, Michael Adamzik, Jürgen Peters, Ulrich H. Frey

**Affiliations:** 1 Klinik für Anästhesiologie, Intensivmedizin und Schmerztherapie, Universitätsklinikum Knappschaftskrankenhaus Bochum, In der Schornau 23-25, D-44892 Bochum, Germany; 2 Klinik für Anästhesiologie und Intensivmedizin, Universität Duisburg-Essen & Universitätsklinikum Essen, D-45122 Essen, Germany

**Keywords:** ARDS, microRNA, miR-122, Acute liver injury; acute liver dysfunction, 30-day mortality, Bilirubin

## Abstract

**Background:**

Acute liver injury in patients with ARDS decreases survival but early stages may be easily missed due to the lack of sufficient biomarkers signalling its onset. Accordingly, we tested in ARDS patients the hypotheses that microRNA-122, the foremost liver-related microRNA (miR), 1) is an sensitive and specific early predictor for potential liver injury and 2) analysed its impact on 30-day-survival.

**Methods:**

We collected clinical data and analysed blood samples from 119 ARDS patients within the first 24 h of ICU admission and from 20 patients undergoing elective abdominal non-liver surgery serving as controls. Total circulating miR was isolated from serum and relative miR-122 expression was measured (using specific probes and spiked-in miR-54), as were liver function and 30-day survival. Acute liver injury was defined as a total bilirubin concentration ≥ 3.0 mg/dl, an ALT activity ≥350 U/l, and an INR ≥2.0.

**Results:**

30-day survival of the entire ARDS-cohort was 69% but differed between patients with normal liver function (77%) and acute liver injury (19% *p* <  0.001). miR-122 expression was 20fold higher in non-survivors (95%-CI 0.0149–0.0768; *p* = 0.001) and almost 4fold greater in survivors (95%-CI: 0.0037–0.0122; *p* = 0.005) compared to controls (95%-CI 0.0008–0.0034) and correlated with markers of liver cell integrity/function [ALT (*p* <  0.001, *r* = 0.495), AST (*p* <  0.001, *r* = 0.537), total bilirubin (*p* = 0.025, *r* = 0.206), INR (p = 0.001, *r* = 0.308), and GLDH (*p* <  0.001, *r* = 0.489)]. miR-122 serum expression discriminated survivors and non-survivors (AUC: 0.78) better than total bilirubin concentration (AUC: 0.66). Multivariable Cox-regression analysis revealed both acute liver injury (HR 7.6, 95%-CI 2.9–19.8, *p* <  0.001) and miR-122 (HR 4.4, 95%-CI 1.2–16.1, *p* = 0.02) as independent prognostic factors for 30-day mortality.

**Conclusions:**

Increased miR-122 serum expression is an early and independent risk factor for 30-day mortality in ARDS patients and potentially reveal an acute liver injury earlier than the conventional markers of liver cell integrity.

**Electronic supplementary material:**

The online version of this article (10.1186/s12871-018-0541-5) contains supplementary material, which is available to authorized users.

## Background

Acute respiratory distress syndrome (ARDS) is characterized by a severe destruction of the pulmonary parenchymal integrity with consecutive hypoxemia, decreased lung compliance, and diffuse bilateral pulmonary infiltrates and still associated with a high mortality, especially for its severe forms [1, 2]. While the understanding of its underlying risk factors and pathophysiological mechanisms has significantly improved, ARDS-progression is often associated with acute organ-injuries and organ-dysfunctions that finally determine outcome and mortality. In particular, acute liver dysfunction often complicates disease progression and is highly associated with a poor prognosis [3–5].

Diagnostic options refer to the early detection of an acute liver injury, also in patients suffering from ARDS, and comprise of standard biochemical liver tests such as transaminase activity, bilirubin concentration, or markers for liver related synthesis like the liver dependent coagulation factors [6]. In this context, bilirubin concentration is a predictor for ARDS outcome, and indicates acute liver dysfunction in ARDS patients [5]. Nevertheless, all these tests have a low specificity to determine the presence of acute liver injury in its early phase [7–9] implying a need for reliable biomarkers for an early prediction.

MicroRNA’s (miRs) are a diverse family of molecules with 21–24 nucleotide length that regulate RNA expression at the post-transcriptional level [10]. Circulating miRs are protected against degradation by inclusion in extracellular microvesicles [11] or by formation of protein-microRNA complexes with Argonaute 2 [12], nucleophosmin [13], or HDL [14]. Therefore, circulating miRs are remarkably stable, can be quantified, and their importance as biomarkers for various disease states has been established and extensively reviewed [15, 16].

Circulating miR-122 is described as a tissue-specific RNA that represents almost 70% of all liver miRs and only minimally expressed in other tissues [17]. Influencing hepatocyte homeostasis by controlling cellular differentiation, proliferation, and apoptosis via several genes, miR-122 was shown to be a specific serum biomarker for hepatocyte damage [18, 19]. Compared to an increase in blood alanine aminotransferase activity (ALT), increased miR-122 concentration occurred earlier and its magnitude of change was more specific and more sensitive to indicate acute liver injury, and showed a better correlation with liver histology [20]. Considering the extensive literature on miRNA-122, showing a direct association with a hepatic cellular damage and acute liver injury respectively in early stages [21–23], miR-122 might be also an useful biomarker for predicting mortality and an acute liver injury in ARDS patients.

Accordingly, we tested in ARDS patients the hypotheses that microRNA-122, 1) is an sensitive and specific early predictor for acute liver injury and 2) analysed its impact on 30-day-survival.

## Methods

This study was reviewed and approved by the Ethics Committee of the Medical Faculty of the University of Duisburg-Essen (01–97-1697) and informed consent was obtained from patients or their guardians. Patients admitted to the intensive care unit (ICU) of University of Duisburg-Essen Medical School between 2002 and 2011 were considered eligible if they fulfilled the joint American/European Consensus Committee criteria for ARDS [24], had no previous history of ARDS, and did not suffer from any type of chronic liver disease. In addition, we tested all patients whether they fulfilled the formal criteria of the current ARDS definition and no patients had to be excluded due to the current definition [1, 2]. Patients were treated with a multimodal concept, which included analgesia and sedation, fluid administration, lung-protective mechanical ventilation, anticoagulation, as well as hemodynamic, antibiotic, and diagnostic management as described previously [25]. In total, 119 patients with ARDS (71 males (60%), 48 females (40%), mean age: 43.7 years ±13.3) were included in this retrospective analysis. Many patients were referred from other ICU’s for possible extracorporeal membrane oxygenation (ECMO) therapy following a rapidly progressive ARDS course. ARDS was evoked in 104 cases (87%) by infectious pneumonia (bacterial *n* = 76, 64%; H1N1-infection *n* = 28, 23%), in 8 cases (7%) by extrapulmonary sepsis, and in 7 cases (6%) by other reasons.

Blood samples for later analysis were drawn within the first 24 h, normally immediately at admission and before the start of an ECMO-therapy or hemofiltration/−dialysis. Only in 5 cases, the ECMO-therapy was already initiated in the external hospital and blood samples used for miR-122 determination were obtained afterwards. Additionally, a large body of clinical and demographic data was entered into a data base and analysed retrospectively, including pre-existing morbidities, Lung Injury Score, Simplified Acute Physiology Score II, Sepsis-related Organ Failure Assessment Score (SOFA), body mass index, necessity for continuous hemofiltration/−dialysis, mode and settings of mechanical ventilation, PaO2/FiO2 ratio (Horowitz Index), establishment of extracorporeal membrane oxygenation (ECMO) therapy, expanded vital parameters (heart rate, blood pressure, pulmonary artery pressure, cardiac index, stroke volume, etc.), medications and dosage of vasoactive drugs and blood chemistry values. This also included markers of infection (C-reactive protein, procalcitonin, and white blood cell concentrations) and markers of liver cell integrity/function (such as aspartate transaminase: AST; alanine aminotransferase: ALT; glutamate dehydrogenase: GDH), of cholestasis (total bilirubin, direct bilirubin, gamma glutamyltransferase: GGT, alkaline phosphatase: ALP), and those of liver synthetic function (prothrombin time). The observation period was defined from admission to our ICU either to day 30 of hospital stay or death. The ARDS patients were assigned to two groups (with vs. without acute liver injury) depending on the fact of whether they met the formal criteria for an acute liver injury at least on one day in the 30-day observation period. In this context, acute liver injury was defined as a total bilirubin concentration ≥ 3.0 mg/dl, an ALT activity ≥350 U/l, and an INR ≥2.0 [26]. Clinical characteristics of this ARDS cohort are presented in Table 1.

**Table 1:** Baseline characteristic of survivors and non-survivors (30 days)

Variable	Survivors *n* = 82 (69%)	Non-survivors *n* = 37 (31%)	*P*-value
Age *yrs.* (range/± SD)	42.1 (18–70/±14.5)	47.2 (22–64/±10.8)	0.055
Male gender (%)	50 (61)	21 (57)	0.664
Aetiology of ARDS			0.663
- Pneumonia (other than H1N1)	51 (62%)	25 (68%)	0.572
- H1N1-Infection	19 (23%)	8 (21%)	0.852
- Extrapulmonary Sepsis	7 (9%)	1 (3%)	0.239
- Other	5 (6%)	3 (8%)	0.925
Body mass index (*kg/m*^*2*^)	27.7 (±6.3)	26.8 (± 5.6)	0.440
Mean pulmonary artery pressure (*mmHg)*	35.0 [30–39]	36 [28–39]	0.961
Pulmonary vascular resistance index (dyn*s/cm^5^*m^*2*^*)*	333 [206–472]	318 [205–453]	0.942
C-reactive protein concentration (*mg/dl)*	26.3 [14.8–34.7]	20.3 [13,6–28.2]	0.510
Procalcitonin concentration (*ng/m*l)	1.8 [0.6–11.4]	8.3 [1.1–48.3]	0.012
Leukocyte concentration (*10^*9*^*/l*)	14.7 [8.6–21.6]	12.1 [8.8–20.6]	0.416
AST activity (*U/l)*	68.5 [37.0–134,3]	85.5 [66.5–293.0]	0.001
ALT activity (*U/l)*	39.0 [23.8–63.3]	54.0 [25.5–106.5]	0.006
Total bilirubin concentration (*mg/dl)*	0.6 [0.4–1.3]	1.5 [0.7–3.4]	< 0.001
GLDH activity (*U/l*)	6.6 [3.3–11.7]	20.7 [6.5–111.7]	0.003
INR	1.2 [1.0–1.5]	1.4 [1.0–1.7]	0.071
Platelet Count *(/nl)*	171 [115–249]	70.5 [42.5–159]	0.002
PTT *(s)*	38 [33–63]	66 [45–100]	0.033
ECMO therapy (%)	21 (28)	11 (33)	0.548
Dialysis (%)	39 (58)	21 (68)	0.172
Cardiovascular disease (%)	11 (13)	7 (19)	0.428
Prior lung disease (%)	11 (13)	6 (16)	0.686
SAPS II	47.7 (±20.8)	53.8 (±16.9)	0.158
Lung injury score	3.2 (±0.56)	3.2 (±0.52)	0.507
SOFA	12.5 (±6.2)	15.3 (±6.0)	0.025
Mean Airway Pressure *(mmHg)*	26.5 [23.5–29.6]	27.3 [24.2–30.4]	0.285
Horowitz-index upon ICU admission	112.5 [73–191.5]	90.5 [65.5–204.3]	0.295

Data are presented as n (%); mean (± SD) median (25th, 75th percentile), *AST* Aspartate aminotransferase, *ALT* Alanine aminotransferase, *GLDH* Glutamate dehydrogenase, *INR* International Normalized Ratio, *PTT* Partial thromboplastin time, *ECMO* extracorporeal membrane oxygenation, *SAPS II* Simplified Acute Physiology Score, *LIS* Lung-Injury-Score, *SOFA* Sepsis-related Organ Failure Assessment score; Horowitz-Index: paO2/FiO2

### Control patients

Twenty adult patients (9 men, 11 women, mean age 47.9 years ±20.2) without ARDS and without liver disease undergoing elective extrapulmonary and extrahepatic surgery served as controls following informed consent. They were free of lung, cardiac, infectious, and allergic diseases. 5 patients with solid tumours were included (colorectal cancer, *n* = 3; ovarian tumour, *n* = 1; and cervical cancer, n = 1). 12 patients were formally healthy, without any prescribed chronic diseases. Blood sampling was performed by peripheral venous aspiration immediately following induction of anaesthesia.

### Blood sample collection, preparation, and storage

Two blood samples were collected in Vacuette® Serum Tubes and processed within 30 min. Following centrifugation for 10 min at 2500 rpm the serum samples were shock frozen in liquid nitrogen and stored at − 80 °C until analysis. All samples were thawed altogether for the first time for miRNA analysis to circumvent possible degradation from multiple freezing and thawing procedures.

### Serum microRNA extraction

miR was isolated from 400 μl serum using the miRVana PARIS Kit (Ambion, Life Technologies Corporation, USA) according to the manufacturer’s instructions. Due to the lack of validated reference miRs for normalization, 25 fmol exogenous cel-miR-54 from *C. elegans* (Qiagen, Hilden, Germany) was spiked into samples immediately before miR isolation, as described previously [27]. This allows adjustment for differences in sample preparation efficacies. Total RNA was eluted in 100 μl of RNase-free water and stored at − 80 °C until further use.

### Measuring miR-122 expression by quantitative PCR (qPCR)

Relative quantification of miR-122 was carried out using the a qRT-PCR miR Detection Kit (Ambion®, Life Technologies, USA) using hsa-miR-122 PCR Primer Sets for amplification of the miR-122. qPCR was performed using the Applied Biosystems Step One Plus Real-Time PCR System taking advantage of the Taqman miR Assays for cel-miR-54, miR-122, and the Taqman Universal Master Mix II no UNG (all Applied Biosystems, Carlsbad, USA) in a final volume of 20 μl including 1 μl cDNA from the RT reaction as template. All samples were run in duplicate. PCR conditions were as follows: incubation of the samples for 10 min at 95 °C followed by application of 40 cycles of 15 s at 95 °C and 1 min at 60 °C. Relative expression of miR-122 with cel-miR-54 as control was expressed using the comparative CT method using 2^-ΔCT^ [28]. Moreover, miRNA quality was not influenced by duration of storage since all samples were analysed in duplicate and no relevant deviation was detectable (*p* = 0.897). Finally, regression analysis with storage duration and miR-122 expression revealed no significant association of storage duration and miRNA-122 expression (*p* = 0.526).

### Statistical analyses

Continuous variables are presented as means ± standard deviation (SD) in case of normal distribution and as median and interquartile range (25th; 75th percentile) in case of non-normally distributed variables. Categorical variables were characterized by numbers with percentage and were compared using the Chi-square test. Continuous variables were compared using parametric ANOVA (including Bonferroni Holm post hoc testing) or non-parametric Kruskale-Wallis statistics (followed by post-hoc Dunn’s Test). Correlation between miR-122 expression and other variables was analysed using the Spearman correlation test, and values of *p* <  0.05 were considered statistically significant.

Predictive validity of ALT, bilirubin and INR of day 1, 5, and 10 as well as miR-122 expression regarding 30-day mortality were assessed with receiver operator characteristics (ROC) and corresponding results for area under the curve (AUC). In a second step, ROC-analysis was used to define miR-122 cut-off values with the highest sum of sensitivity and specificity to discriminate between survivors and non-survivors. Reclassification analyses using net reclassification improvement (NRI) and integrated discrimination improvement (IDI) were used to assess the added value of miR-122 to the SOFA-score for prediction of 30-day mortality. Furthermore, 30-day survival was displayed using Kaplan-Meier plots with univariate log-rank test for trend. A multivariable Cox-regression analysis including the variables with *p* <  0.1 from univariate demographic statistics was used to determine whether acute liver injury and categorized miR-122 expression were independently associated with 30-day survival. Variables showing significant collinearity were excluded from each model. To avoid overfitting, a restricted model was assessed afterwards with only three (Table 4) and four variables (Table 5), respectively, using only those predictors with a *p*-value of ≤0.10. Hazard ratios (HR) and 95% confidence intervals (CI) were calculated from the Cox regression analysis to describe the effect of covariates on the hazard. All analyses were performed using SPSS (version 24, IBM, Chicago, IL, USA) and for graphical presentations GraphPad Prism 7 (Graph-Pad, San Diego, CA, USA) was used.

## Results

**Fig. 1:**
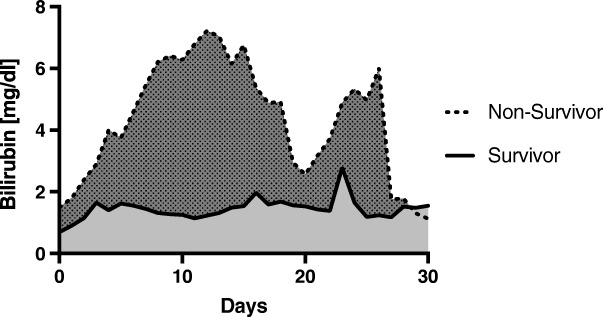
Total serum bilirubin concentration [mg/dl] depicted over a period of 30 days after admission to ICU

Table 1 shows the baseline characteristics upon admission of the 119 ARDS-patients. The observed 30-day survival was 69% and median duration of ICU stay was 19 days [13; 34 days]. The patients demonstrated a SOFA-Score at admission of 13.4 (± 6.3) with a significant difference between survivors (12.5 ± 6.2) and non-survivors (15.3 ± 6.0; *p* = 0.025). ARDS patients were severely hypoxemic on inclusion, with a Horowitz-Index of 102 mmHg [73–190 mmHg], a Lung-Injury-Score of 3.2 (± 0.55) and 116 of our 119 ARDS-patients (97.5%) were mechanically ventilated on day one. Venovenous extracorporeal gas exchange was necessary in 32 of 119 patients (37%), and haemodialysis/hemofiltration was established in 60 patients (50%) within the 30-day observation period. Concerning markers of liver cell integrity, AST, ALT, bilirubin, and GLDH were all significantly increased in ARDS non-survivors upon admission (Table 1) with bilirubin concentration showing a peak of 7.2 mg/dl in non-survivors on day 12. This was 5-fold greater compared to survivors (*p* <  0.001, Fig. 1).

**Fig. 2:**
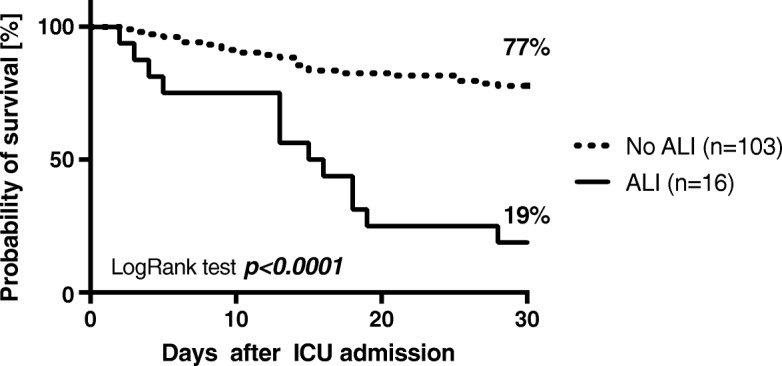
Thirty-day survival in patients with the acute respiratory distress syndrome (ARDS) stratified for patients with and without acute liver injury (ALI), defined as a total serum bilirubin concentration ≥ 3 mg/dl, an ALT activity ≥350 U/I, and an INR ≤2.0. Kaplan-Meier estimates were used to calculate probabilities of 30-day survival. Thirty-day survival was 77% in patients not suffering from acute liver injury but only 19% in ARDS patients with an ALI (*p* < 0.001)

None of the patients fulfills the formal criteria for an acute liver injury at admission to our ICU [26]. The incidence of acute liver injury within the 30 day observation period was 13% (*n* = 16) according to the criteria of the Acute Liver Failure Study Group [26] and Kaplan-Meier survival analysis showed that ARDS patients suffering from acute liver injury had an almost 4-fold greater mortality (81%) than ARDS patients without acute liver injury (23%; *p* <  0.001, Fig. 2).

**Fig. 3:**
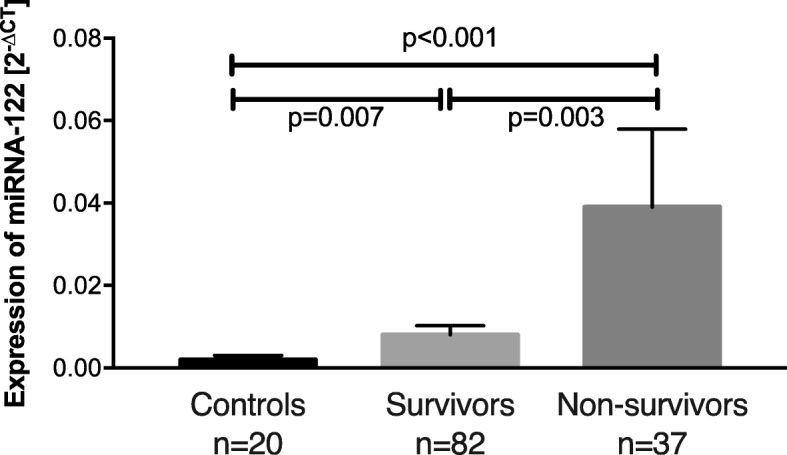
Relative miR-122 expression (2^-ΔCT^) in control patients, ARDS survivors, and ARDS non-survivors. Means and standard error of the mean of miR-122 expression

The relative expression of miR-122 was 9-fold increased in patients suffering from ARDS compared to controls (*p* <  0.001). Within our control-group miR-122-expression was not different between patients with or without tumour disease (*p* = 0.205). Furthermore, we found a 20-fold increased miR-122 serum expression in ARDS non-survivors (*p* < 0.001) compared to an only 4-fold greater miR-122 expression in ARDS survivors (*p* = 0.007) compared to controls (Fig. 3). Comparing ARDS survivors with non-survivors, we found an almost 5-fold greater miR-122 expression in ARDS non-survivors (*p* = 0.003; Fig. 3). Regarding the specificity of miR-122 for an acute liver failure, miR-122 concentrations were 7-fold higher in survivors with acute liver injury (*p* = 0.022) and 6-fold increased in non-survivors with acute liver injury (*p* = 0.002) compared to survivors and non-survivors without an acute liver injury, respectively (Additional file 1). We next analysed the impact of acute liver injury and other variables on miR-122 expression by performing correlation analyses with laboratory markers and hemodynamic values. In these analyses, miR-122 expression strongly correlated with other established markers of liver damage such as AST (*p* < 0.001), ALT (*p* < 0.001), LDH (*p* < 0.001), and GLDH (*p* < 0.001). Slightly weaker but still significant correlations were identified with classic liver function markers such as total bilirubin (*p* = 0.025), direct bilirubin (*p* = 0.003), and INR (*p* = 0.001; Table 2). In contrast, we found no significant correlation of miR-122-expression and markers of cholestasis such as AP and GGT (Table 2).

**Table 2:** Correlation between relative miRNA-122 expression (ΔCT) with other laboratory markers and haemodynamic variables

		*p* value	Spearman’s correlation coefficient (r)
Liver damage			
- AST activity		< 0.001	0.537
- ALT activity	Day 1	< 0.001	0.495
	Day 5	< 0.001	0.523
	Day 10	0.004	0.288
	Day 15	0.003	0.329
	Peak_30day_	< 0.001	0.490
- GLDH activity		< 0.001	0.489
- LDH activity		< 0.001	0.494
Cholestasis			
- GGT activity		0.659	
- AP activity		0.610	
Liver function			
- Bilirubin concentration	Day 1	0.025	0.206
	Day 5	0.032	0.204
	Day 10	0.496	
	Day 15	0.641	
	Peak_30day_	0.070	
- Direct bilirubin concentration		0.003	0.278
- INR	Day 1	0.001	0.308
	Day 5	0.017	0.227
	Day 10	0.155	
	Day 15	0.337	
	Peak_30day_	0.002	0.284
Inflammation			
- C-reactive protein concentration		0.872	
- Procalcitonin concentration		0.274	
- Interleukin-6 concentration		0.132	
- TNF-Alpha concentration		0.425	
- White blood cell count		0.720	
Cardiovascular function			
- Cardiac Index		0.386	
- Stroke Volume Index		0.369	
- Systemic Vascular Resistance Index		0.271	
- Mid arterial pressure		0.001	−0.320
Right heart burden			
- Mid pulmonary artery pressure		0.886	
- Central venous pressure		0.533	

*ALT* Alanine aminotransferase, *AST* Aspartate aminotransferase, *GLDH* Glutamate dehydrogenase, *LDH* Lactate dehydrogenase, *GGT* Gamma-glutamyl transferase, *AP* alkaline phosphatase, *TNF* Tumour necrosis factor; Peak_30day_: Peak value of 30 day oberservation period; not labelled measurements were taken on day 1

To analyse potential triggers for acute liver injury and miR-122 expression we performed correlation analyses between miR-122 expression and markers of inflammation (CRP, PCT, IL-6, TNF- α, WBC), global cardiovascular function (CI, SVI, SVRI, MAP), and right heart burden (CVP, mPAP). miR-122 expression was not correlated with markers of inflammation or right heart burden (Table 2). However, miR-122 expression showed a correlation with some values of global cardiovascular function, including a significant negative correlation with mean arterial pressure (*p* = 0.001, Table 2). Furthermore, we did not find any correlation between the expression of miRNA-122 and PEEP (*p* = 0.207, Table 3), mean airway pressure (*p* = 0.633) or ECMO therapy (*p* = 0.303). In addition, miR-122 concentration of the 5 patients with ECMO-therapy, initiated before taking the first blood sample upon ICU-admission, showed no significant difference compared to the other patients of entire cohort (*p* = 0.484). In case of a renal replacement therapy we found significant higher miR-122 serum levels (*p* = 0.029).

**Table 3:** ROC statistics for miR-122 and serum bilirubin concentration over the course of disease and treatment

Test	AUC	Confidence interval	Significance level
miR-122	0.782	0.694–0.870	< 0.001
Bilirubin conc. on day 1	0.663	0.564–0.763	0.003
Bilirubin conc. on day 5	0.655	0.546–0.764	0.007
Bilirubin conc. on day 10	0.767	0.662–0.871	< 0.001
ALT activity on day 1	0.574	0.427–0.720	0.188
ALT activity on day 5	0.476	0.326–0.626	0.732
ALT activity on day 10	0.534	0.383–0.686	0.630
INR on day 1	0.551	0.412–0.690	0.470
INR on day 5	0.603	0.464–0.758	0.185
INR on day 10	0.684	0.561–0.808	0.010

Area under curve, asymptotic confidence interval as well as asymptotic *P*- values are given for relative miR-122 expression, serum bilirubin concentration Alanine aminotransferase (ALT) activity in U/l, and International Normalized Ratio (INR) on day 1, 5, and 10

**Fig. 4:**
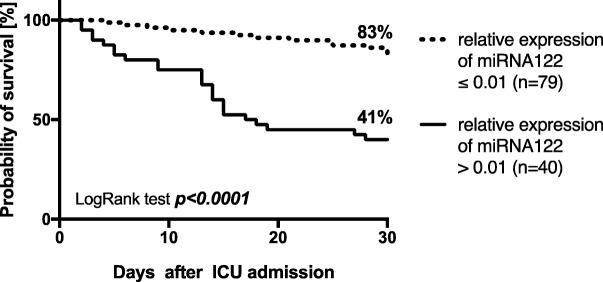
Thirty-day survival in patients with the acute respiratory distress syndrome (ARDS) stratified for relative miR-122 expression (2^-ΔCT^) in a relative expression rate in serum ≤0.01 and > 0.01. Kaplan-Meier estimates were used to calculate probabilities of 30-day survival. Thirty-day survival was 83% in patients with a 2^-ΔCT^ ≤ 0.01 but only 41%in patients with a relative miR-122 expression of 2^-ΔCT^ > 0.01 (*p* < 0.001)

The prognostic value of miR-122 expression to predict 30-day mortality was evaluated using receiver-operating analysis and revealed a cut-off ratio of 0.01, as the value with the highest sum of sensitivity and specificity, to discriminate between ARDS survivors and non-survivors. miR-122 expression revealed a sufficient discrimination (AUC: 0.78; *p* < 0.001) and a cut-off expression of 2^-∆CT^ of 0.01 resulted in a sensitivity of 61%, a specificity of 86%, a negative predictive value of 0.84 and a positive predictive value of 0.63. Using this cut-off, 30-day survival could be calculated as 83% for patients with a 2^-∆CT^ ≤ 0.01 but only 41% for patients with a 2^-∆CT^ > 0.01 (*p* < 0.001, Fig. 4).

**Fig. 5:**
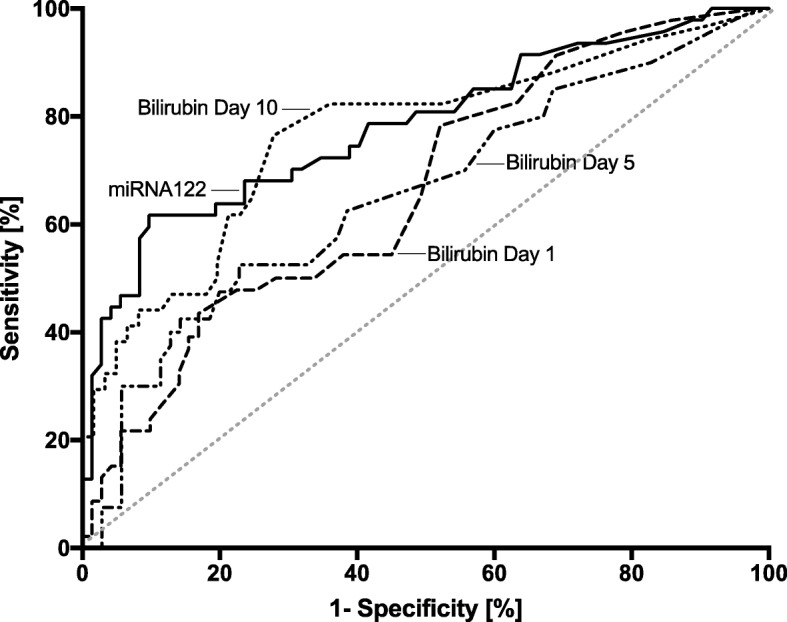
Relative miR-122 expression (2^-ΔCT^) in serum and total serum bilirubin concentrations on day 1, 5, and 10 in patients with ARDS. Shown are the receiver operating characteristics. Comparing total bilirubin concentrations on day one (AUC 0.663), day five (AUC 0.655), and day 10 (AUC 0.767) with miR-122 (AUC 0.782).Only total bilirubin concentration on day 1 reached a comparable reliability

To further compare the value of circulating miR-122 expression as biomarker with the criteria for acute liver injury proposed by the Acute Liver Failure Study Group of UTSWMC [26] receiver operating characteristic curves were generated for total bilirubin concentration, ALT, and INR on days 1, 5, and 10 (Table 3, Fig. 5). Comparing variables of day one with miR-122 expression the AUCs as a measure of assay reliability were 0.782 for miR-122 (*p* < 0.001), 0.663 for total bilirubin concentration (*p* = 0.003), 0.574 for ALT activity (*p* = 0.188), and 0.551 for INR (*p* = 0.47). Only bilirubin concentration on day 10 reached a comparable reliability with a AUC of 0.767 (*p* < 0.001, Fig. 5). The other AUC’s of day 5 and 10 as well as the significance levels and the confidence intervals are shown in Table 3. Furthermore, the predictive value for 30-day mortality of the SOFA-Score (AUC 0.702) was improved by adding miR-122 (AUC 0.748; NRI 0.271, *p* = 0.016; IDI 0.04, *p* = 0.037).

Multivariable Cox-Regression referring to acute liver injury including as covariates age, SOFA-Score, platelet count, GLDH and AST activities, PTT, and procalcitonin concentration revealed acute liver injury as a major, important, and independent prognostic factor for 30-day survival both in the initial (HR 8.4; 95%-CI 3.0–23.5; *p* < 0.001) and the restricted model (HR 7.7; 95%-CI 3.0–19.8; *p* < 0.001) along with age and platelet count (Table 4). The second cox-regression analysis referring miR-122 expression to age, SOFA-Score, platelet count, bilirubin concentration, AST and GLDH activities, INR, PTT, and procalcitonin concentration as covariates also exposed age and platelet count as important prognostic factors. Furthermore, patients with miR-122 expression above the cut-off of 0.01 had a hazard ratio of 5.4 in the initial model (95%-CI 1.3–22.6, *p* = 0.021) and a HR of 4.4 (95%-CI 1.2–16.1, *p* = 0.016) in the restricted model and, therefore, showed significant impact on 30-day survival (Table 5).

**Table 4:** Cox regression analysis in patients with ARDS referring ALI-definition of the Acute Liver Failure Study Group of the UTSWMC

(Co) variable	Multivariate					
	Initial			Restricted		
	*p*-value	HR	95%- CI	*p*-value	HR	95%- CI
Patients without ALI	–	1		–	1	
Patients with ALI	< 0.001	8.377	2.994–23.436	< 0.001	7.645	2.959–19.756
Age [yrs.]	0.073	1.039	0.996–1.084	0.015	1.048	1.009–1.089
SOFA score [per unit]	0.457	1.023	0.964–1.085			
Platelet count [10^9^/l]	0.094	0.995	0.989–1.001	0.045	0.995	0.990–1.000
GLDH activity [U/l]	0.898	1.000	0.998–1.001			
AST activity [U/l]	0.312	1.000	1.000–1.001			
PTT activity [s]	0.534	1.004	0.992–1.015			
Procalcitonin concentration [ng/ml]	0.350	0.999	0.998–1.001			

*HR* Hazard ratio point estimates, 95% CI, and *p*-values (two-sided) from Wald tests are reported, *ALI* Acute Liver Injury according the study inclusion criteria of the Acute Liver Failure Study Group of the UTSWMC, *SOFA* Sepsis-related Organ Failure Assessment score, *GLDH* Glutamate dehydrogenase, *AST* Aspartate aminotransferase, *PTT* Partial thromboplastin time

**Table 5:** Cox regression analysis in patients with ARDS referring relative expression of miR-122

(Co) variable	Multivariate							
	Initial					Restricted		
	*p*-value	HR	95%- CI			*p*-value	HR	95%- CI
miR-122 ≤ 0.01 [2^-∆CT^]	–	1				–	1	
miR-122 > 0.01 [2^-∆CT^]	0.021	5.392	1.285–22.619			0.016	4.441	1.197–16.110
Age [yrs]	0.033	1.048	1.004–1.095			0.013	1.047	1.010–1.087
SOFA score [per unit]	0.354	1.029	0.969–1.092					
Platelet count [10^9^/l]	0.065	0.992	0.984–1.000			0.002	0.991	0.985–0.997
Bilirubin concentration [mg/dl]	0.074	1.299	0.975–1.730			0.103	1.239	0.957–1.603
AST activity [U/l]	0.121	1.002	0.999–1.004					
GLDH activity [U/l]	0.646	1.001	0.998–1.003					
INR	0.404	1.012	0.991–1.034					
PTT [s]	0.574	1.004	0.989–1.020					
Procalcitonin concentration [ng/ml]	0.737	1.000	0.998–1.003					

*HR* Hazard ratio point estimates, 95% CI, and *p*-values (two-sided) from Wald tests are reported, 2^-∆CT^: relative expression level of miRNA 122, *SOFA* Sepsis-related Organ Failure Assessment score, *AST* Aspartate aminotransferase, *GLDH* Glutamate dehydrogenase, *PTT* Partial thromboplastin time

## Discussion

This study, to our knowledge, is the first to assess whether miR-122 serum levels are associated with altered survival and indicates of an acute liver injury in patients suffering from ARDS. We demonstrated that miR-122 is an independent risk factor for 30-day survival and its serum concentration is almost 5-fold higher in ARDS non-survivors compared to survivors. Accordingly, our results reveal that increased miR-122 serum levels seems to be an early and reliable biomarker to detect patients with an acute liver injury and therefore could help to indicate an increased mortality risk in patients suffering from ARDS in an earlier disease stage.

Considering multiple organ dysfunction, early liver dysfunction is independently associated with a worse outcome in patients suffering from ARDS [5, 29, 30] In other critically ill patients, liver injury also occurs frequently during the ICU stay and is strongly related to ICU mortality [3, 4]. Diagnostic options normally incorporate analysis of standard liver tests such as transaminase activity, bilirubin concentration, or markers for liver related synthesis like the liver dependent coagulation factors [6]. According to this, the Acute Liver Failure Study Group of the University of Texas Southwestern Medical Centre (UTSWMC) defined criteria for acute liver injury in the absence of measurable hepatic encephalopathy or non-acetaminophen aetiology as acute hepatic illness of < 26 weeks, INR ≥ 2.0, ALT of ≥350 U/I, and total bilirubin concentration of ≥3.0 mg/dl [26]. In this context, we demonstrated a crucial impact of acute liver injury on survival and found significantly greater bilirubin concentrations and ALT activities as well as lesser INR values comparing survivors with non-survivors, in accordance to prior studies on this topic [31].

Studies have suggested that serum bilirubin concentration is a sensitive marker for an acute liver dysfunction and strongly correlates with survival [5, 32]. However, in one study [5], bilirubin concentrations peaked at day 14 after admission while concentrations at admission were less than 2 mg/dl in non-survivors. Corresponding, our receiver operating characteristics revealed, that only bilirubin concentration on day 10 has a reliability comparable to miR-122 expression on day 1. Thus, bilirubin is hardly an early predictor for acute liver injury. Furthermore, other studies suggest that bilirubin concentration is not very specific for acute liver injury and, therefore, likely to underestimate the problem [9, 21].

With respect to other markers of liver cell integrity, prior studies reported that the increase in ALT and AST activities also occur too late for diagnosing an acute liver injury in time, raising a demand for new, reliable, and early biomarkers [20, 21]. Following toxic injury in rodents, serum miR-122 expression increases earlier than ALT activity. This supports the value of miR-122 as an early marker [33]. Consistent with this, the AUC’s of ALT activities on day 1 did not reach the predictive value seen with miR-122. Accordingly, miR-122 used as biomarker may also allow early detection of an acute liver injury, besides prediction of mortality in ARDS patients. To draw definite conclusions and truly validate the value of miR-122 as a marker of liver injury in critical ill patients like ARDS, further prospective and well-designed studies implying tissue biopsies, functional tests or a control group with liver dysfunction without critical illness are needed.

Our study does not pinpoint the mechanisms for increased miR-122 serum levels with acute liver injury. However, since increased miR-122 concentrations indicate a poor neurological outcome in patients after resuscitation from cardiac arrest, organ malperfusion may up-regulate circulating miR-122 [34]. This hypothesis is supported by a significant correlation between miR-122 expression and arterial pressure, suggesting that decreased liver perfusion may trigger acute liver injury and thereby release of miR-122 due to liver cell damage via apoptosis and necrosis. This is further supported by the significant correlation of miR-122 with LDH, a marker of cellular damage in association with acute liver injury. However, microvascular injury and/or disturbed microvascular blood flow and cellular oxygenation with ARDS and sepsis might also play a role. Wang et al. published in 2014 that miR-122 can be used as a biomarker in patients with both sepsis and ARDS and show an association with 28-day mortality in the different ICUs [35]. Additionally, Leelahavanichkul and colleagues reported in an indirect liver injury model in rodents simulating diseases like sepsis and ARDS, that increased miR-122 serum levels may be explained, at least in part, by cytokine accumulation inducing miR-122 production with temporal latency [36]. In contrast to these results, we could not find a correlation between miR-122 expression and markers of inflammation. Results from other experiments in mice and data from septic patients also suggest that miR-122 expression is independent of sepsis [21] and neither a correlation between miR-122 and other inflammatory miRs was observed [21], nor classic inflammatory markers like white blood cell count, CRP or procalcitonin concentration were correlated [21, 34].

Furthermore, the possibility of a complex organ crosstalk must also be considered in critically ill patients, like our ARDS-patients. Here, hepatic injury may be induced by acute renal failure that could occur before breakdown of the intestinal barrier, which eventually results in multiorgan dysfunction [37]. This hypothesis is in line with the observation of higher miR-122 serum levels in cases of hemofiltration or –dialysis.

The crucial issue of mechanisms for stimulating miR-122 expression should be investigated and clarified in further prospective studies.

### Limitations

The enrolled patients represent a more or less unselected cohort of severe ARDS patients and our data may not be representative for other cohorts. Furthermore, undetected confounding factors may have distorted the results. In this context, we cannot exclude an influence on our results due to the different duration of probe storage as potential confounding factor. In addition, the fact that some patients used as controls suffered from chronic diseases, solid tumours necessitating surgery may alter miR-122 expression. However, we did not observe any significant different expression levels between controls with or without a tumour. A further possibly confounding factor is the lower rate of male subjects in our control group (45%) compared to in our ARDS cohort (60%), nevertheless we expect no significant bias in our results from this issue. While repeated miR-122 expression measurements during the time course of ARDS may have expanded the prognostic relationship between acute liver injury and miR-122 expression, associations of miR-122 expression are limited to day 1 predictions. Since we refer to the criteria used by the acute liver failure study group to define an ALI [26], with very restrictive cut-offs, we cannot exclude that choosing less strict cut-off values may improve the prediction value of these indicators. Furthermore, in our study, we can only establish indirect associations between miR-122 Expression and an acute liver injury because histological examinations were not the part of our study. Finally, it is unclear how miR expression should be normalized to account for interindividual or intergroup variability. In our study, we used spiked-in non-human RNAs before miR extraction for normalization of the miR expression level. Nevertheless, no universally accepted standard for miR measurements has been defined so far [15], limiting comparability of different studies. Furthermore, no reliable and clinically useful cut-offs for miR-122 expression are yet established and validated. Therefore, prospective studies are needed before the usage of miR-122 as a biomarker may become feasible in clinical practice.

## Conclusions

In conclusion, increased miR-122 serum levels are a promising biomarker early predicting short-term mortality and further may be associated with an acute liver injury in ARDS patients.

## Additional file

Additional file 1: Relative miR-122 expression of ARDS survivors and ARDS non-survivors without and with acute liver injury, respectively. (DOCX 146 kb)

## Abbreviations

°C: Degree in Celsius
2^-ΔCT^: Expression of microRNA
ALT: Alanine aminotransferase activity
AP: Alkaline phosphatase activity
ARDS: Acute respiratory distress syndrome
AST: Aspartate aminotransferase activity
AUC: Area under the curve
BMI: Body Mass Index
CI: Confidence Interval
conc: Concentration
CRP: C-reactive protein concentration
ECMO: Extracorporeal membrane oxygenation
FiO2: Inspired fraction of oxygen
GGT: Gamma glutamyltransferase activity
GLDH: Glutamate dehydrogenase activity
HR: Hazard ratio
ICU: Intensive Care Unit
IL-6: Interleukin 6
INR: International Normalized Ratio
LDH: Lactate dehydrogenase activity
LIS: Lung Injury Score
MAP: Mean arterial pressure
min: Minimum
miR: Micro ribonucleic acid
mPAP: Mid pulmonary artery pressure
paO2: Arterial partial pressure of oxygen
PCR: Polymerase chain reaction
RNA: Ribonucleic acid
ROC: Receiver operating characteristic
rpm: Rounds per minute
RT-PCR: Real-time polymerase chain reaction
SAPS II: Simplified Acute Physiologic Score II
SD: Standard deviation
sec: Seconds
SEM: Standard error of the mean
SOFA: Sepsis-related Organ Failure Assessment Score
SVI: Stroke Volume Index
SVRI: Systemic Vascular Resistance Index
TNF: Tumour necrosis factor
TUNEL: Terminal deoxynucleotidyl transferase dUTP nick end labelling
ULN: Upper limit normal
UTSWMC: University of Texas Southwestern Medical Centre
WBC: White bloodcell count
