# Supplementary material for: Increased circulating microRNA-122 is associated with mortality and acute liver injury in the acute respiratory distress syndrome
Source: BMC Anesthesiol. 2018 Jun 23;18:75. doi: 10.1186/s12871-018-0541-5 (PMC6015662; doi:10.1186/s12871-018-0541-5)
Supplement: Supplementary file 1 — Relative miR-122 expression of ARDS survivors and ARDS non-survivors without and with acute liver injury, respectively. (DOCX 146 kb) [file 12871_2018_541_MOESM1_ESM.docx]

**Additional file 1 - Figure**

Relative miR-122 expression (2^-ΔCT^) of ARDS survivors and ARDS non-survivors without and with acute liver injury (ALI), respectively. Means ± standard error of the mean.
